# Staphylococcal mastitis in dairy cows

**DOI:** 10.3389/fvets.2024.1356259

**Published:** 2024-05-28

**Authors:** Oudessa Kerro Dego, Jessica Vidlund

**Affiliations:** ^1^Department of Animal Science, University of Tennessee, Knoxville, TN, United States; ^2^East Tennessee AgResearch and Education Center-Little River Animal and Environmental Unit, University of Tennessee, Walland, TN, United States

**Keywords:** bovine staphylococcal mastitis, *Staphylococcus aureus*, non-aureus staphylococci, dairy cow, control, host immune responses, staphylococcal virulence factors, antimicrobial resistance of staphylococci

## Abstract

Bovine mastitis is one of the most common diseases of dairy cattle. Even though different infectious microorganisms and mechanical injury can cause mastitis, bacteria are the most common cause of mastitis in dairy cows. Staphylococci, streptococci, and coliforms are the most frequently diagnosed etiological agents of mastitis in dairy cows. Staphylococci that cause mastitis are broadly divided into *Staphylococcus aureus* and non-aureus staphylococci (NAS). NAS is mainly comprised of coagulase-negative *Staphylococcus* species (CNS) and some coagulase-positive and coagulase-variable staphylococci. Current staphylococcal mastitis control measures are ineffective, and dependence on antimicrobial drugs is not sustainable because of the low cure rate with antimicrobial treatment and the development of resistance. Non-antimicrobial effective and sustainable control tools are critically needed. This review describes the current status of *S. aureus* and NAS mastitis in dairy cows and flags areas of knowledge gaps.

## 1 Introduction

Mastitis is an inflammation of mammary glands usually caused by bacteria. It can also be caused by fungi or occasionally by mechanical injury, resulting in increased milk somatic cell count (SCC) and/or abnormal changes in milk and gland tissue (1). Mastitis incurs huge economic losses to dairy farming worldwide; in the United States (U.S.) dairy industry alone, economic losses are more than $2 billion annually (2–4). Clinical mastitis costs $444 for each case during 30 days in milk (DIM) post-calving (2). *Staphylococcus aureus* and non-aureus staphylococci (NAS) cause mastitis in dairy cows. *S. aureus* is a major contagious mammary pathogen on the U.S. dairy farms and throughout the globe (5). NAS comprises more than 50 different species of coagulase-negative staphylococci (6–9) and some coagulase-positive and coagulase-variable staphylococci (8, 10–16). Approximately 95% of coagulase-positive *Staphylococcus* isolates from bovine mastitis are *S. aureus* (17), and about 15% of NAS have been linked to bovine mastitis (18, 19). *S. chromogenes* is a predominant NAS (19–21) consistently isolated from subclinical mastitis cases (22, 23), cows’ udder, and teat skin (24, 25).

Management-based mastitis control measures have been developed and implemented with mild success in reducing contagious bacteria such as *S. aureus* and *S. agalactiae* (26–28) but limited success due to the application disparities across mastitis management (29). Dependence on antimicrobial drugs to control *S. aureus* and NAS is not sustainable due to limited success (30, 31) and the emergence of bacteria resistant to the commonly used antimicrobial drugs (32, 33).

Currently, one commercial bacterin vaccine is claimed to have some effects against *S. aureus* mastitis in dairy cows in the US. However, studies evaluating the efficacy of this commercial vaccine found no significant difference between vaccinated and unvaccinated control cows (34–36). Another polyvalent commercial bacterin vaccine containing inactivated high biofilm-forming *S. aureus* strain SP 140 and *E. coli* J5 strain is available in Europe and a few other countries for the control of mastitis caused by *S. aureus*, NAS, *E. coli,* and other coliforms in dairy cows. Some efficacy studies on this vaccine concluded that vaccination with the polyvalent bacterin reduced mastitis incidence, severity, and duration (37–39), whereas others concluded that vaccination with the polyvalent bacterin did not induce a significant reduction in staphylococcal intramammary infection (IMI) between vaccinated and unvaccinated groups (40–43). However, Freick et al. (42) found a significantly lower SCC in cows vaccinated with an autogenous vaccine compared to the unvaccinated group. Based on published vaccine efficacy studies in the United States, currently available vaccines cannot be recommended as part of the routine measures for controlling mastitis due to *S. aureus* and NAS in dairy cattle. Therefore, effective and sustainable non-antimicrobial bovine *S. aureus* and NAS mastitis control tools are urgently needed.

## 2 Bovine staphylococcal mastitis

*Staphylococcus* belongs to the family of *Staphylococcaceae* (44–46). Based on the 16S rRNA gene sequence similarity and analysis of overall genome-related indices such as DNA–DNA hybridization, average nucleotide identity, and average amino acid identity analyses, some *Staphylococcus* subspecies were reclassified as novel species. Five *Staphylococcus* species (*S. sciuri*, *S. fleurettii*, *S. lentus*, *S. stepanovicii,* and *S. vitulinus*) were reassigned to the new *Mammaliicoccus* genus (47). Since our focus is on the genus *Staphylococcus*, we did not include the *Mammaliicoccus* genus in this review. Staphylococci are opportunistic commensal or opportunistic environmental bacteria that inhabit the nostrils, mucus membranes, and skin of mammals and birds (15, 48). More than 60 valid species exist in the *Staphylococcus* genus (44, 48, 49). In dairy cattle, mastitis is usually caused by *Staphylococcus aureus* (5) and NAS, which comprises coagulase-negative *Staphylococcus* species (CNS) (6, 19, 21) and some coagulase-positive and coagulase variable staphylococci (8, 50, 51).

Staphylococci are non-motile facultative anaerobic [except *S. saccharolyticus* and *S. aureus* subsp. *anaerobius,* which are anaerobic (48)] cocci that grow in an aggregating grape-like cluster due to perpendicular division planes. They are biochemically positive or negative or variable for coagulase, negative for oxidase, and positive for gram staining and catalase (44, 48, 49). Staphylococci can survive in the environment over an extended period (52, 53). They are usually catalase-positive, but some catalase-negative rare strains have also been reported (54, 55). All *Staphylococcus* species are lysed by lysostaphin except a few rare species (55, 56). Staphylococci have a low G/C content of approximately 27–41% in the chromosomal DNA, and most strains grow at 10% NaCl (48). Some species of staphylococci produce coagulase (Coa) and/or von Willebrand factor binding protein (vWbp), both of which can bind to prothrombin and convert it to a complex that can convert fibrinogen in the blood to fibrin (57–59). Coagulase-positive *S. aureus* is considered a major pathogenic species (15, 48, 55), whereas NAS are considered minor pathogens (15, 48, 55). Though a majority of coagulase-positive *Staphylococcus* species from bovine mastitis are *S. aureus* (17), non-aureus coagulase-positive or variable staphylococci occasionally cause mastitis and other diseases in animals, including dairy cows. *Staphylococcus intermedius*, *S. pseudintermedius,* and *S. coagulans* are coagulase-positive *Staphylococcus* species that cause different diseases in dogs and cats and occasionally rare or sporadic cases of bovine mastitis (10–12). *S. aureus* subs. *Anaerobius* (newly reclassified as *S. aureus*) is coagulase-positive and causes chronic purulent subcutaneous inflammation near superficial lymph nodes in sheep and goats (16, 60). Some coagulase variable species (*S. hyicus* and *S. agnetis*) cause mastitis in dairy cows (8). *Staphylococcus hyicus* causes different diseases in pigs (13–15). Some studies reported the presence of atypical strains of *S. chromogenes* that cause clotting of plasma (61).

There are also coagulase-negative variants of *S. aureus* (62, 63). Some coagulase-negative *Staphylococcus* species (*S. chromogenes, S. simulans, S. xylosus*, *S. haemolyticus, and S. epidermidis*) (8, 18, 19) are increasingly reported as the cause of subclinical mastitis and some clinical mastitis in dairy cows (8, 19, 64). The NAS comprises diverse species that vary in pathogenicity, epidemiological distribution, and genomic composition. Describing each species individually and studying its virulence, pathogenicity, distribution, effect on milk somatic cell count (SCC), and milk production losses is more helpful for controlling mastitis caused by these groups of bacteria.

### 2.1 *Staphylococcus aureus* mastitis

*Staphylococcus aureus* is a major contagious mastitis pathogen in the US dairy farms and throughout the globe (5, 65, 66). There are different *S. aureus* strains (67–69) that also vary in their ability to spread in herds (70, 71), cause mastitis (72–74), incur losses in milk yield (75), possess virulence traits (76–78), and invade mammary epithelial cells (79, 80), but a single strain is reported to predominate in a herd (72). Some dominant clones are reported to cause mastitis worldwide (71, 81–83). Campos et al. reported that genotypes CC97, CC1, CC5, CC8, and CC398 are the most predominant lineages isolated from dairy herds worldwide (71). Of these, CC97 and CC151 seem more pathogenic than others based on molecular and genomic comparative analysis (84). A study on *S. aureus* isolates from clinical and subclinical cases of mastitis in Finland found five clonal complexes, including CC97, CC133, CC151, CC479, and CC522 (85). The authors evaluated the presence of a total of 296 virulence factors and found 219 were present in all isolates (85). The authors concluded that there was no association between the presence of virulence factors and clinical outcomes of infection, but the presence of virulence factors varied with clonal complexes (85).

*Staphylococcus aureus* usually causes subclinical mastitis (SCM) and chronic mastitis with high SCC (30, 86). There are considerable variations in the mastitis caused by *S. aureus,* ranging from the peracute form with the development of gangrene in the udder, which usually occurs during early lactation, to more common subclinical chronic forms resulting in increased SCC and decreased milk production (87, 88). In general, *S. aureus* mastitis decreased in farms that fully applied mastitis control programs. In dairy farms with low bulk tank milk SCC, the cow-level prevalence of *S. aureus* IMI is 1–10%. However, in farms with high bulk tank milk SCC, the cow-level prevalence of *S. aureus* IMI may increase to 50–75% with individual udder quarter IMI prevalence of 10–25% (89, 90). The prevalence of *S. aureus* IMI in heifers is 5–15% at parturition (89, 91). *Staphylococcus aureus* mastitis treatment with antibiotics is not effective, and the cure rate is very low (30).

### 2.2 Mastitis due to non-*aureus* staphylococci

#### 2.2.1 NAS as minor pathogens/commensals in the mammary glands

NAS is a group of over 50 different species of coagulase-negative staphylococci, along with some coagulase-positive and variable staphylococci. Despite the presence of different species, only about 15–20 species are associated with bovine IMI, and the most frequent isolates include *S. chromogenes, S. simulans, S. xylosus*, *S. haemolyticus, and S. epidermidis* (8, 18, 19). NAS are increasingly reported as the most frequent isolates from lactating dairy cows (6, 20, 25, 92). Some NAS are frequently reported as etiology of subclinical mastitis in dairy ruminants (6–9, 93, 94), while others occasionally cause mastitis in dairy cows as well as other diseases in animals (10–15). Some studies reported *S. chromogenes, S. simulans*, *S. epidermidis,* and *S. xylosus* as major isolates from teat skin and teat tips, whereas other studies identified *S. chromogenes*, *S. haemolyticus*, and *S. xylosus* as major isolates from milk samples (95–98). *S. chromogenes* usually colonize the skin of teat and udder in heifers during calving (24, 99, 100), bovine milk of primiparous cows during first lactation (101, 102), and milk of cows with mastitis, especially primiparous cows (25, 101–103). *S. simulans* is usually isolated from the milk of cows with mastitis (101, 104–106). *S. agnetis* is a coagulase variable (107) species originally isolated from cows with mastitis and very similar to *S. hyicus* (105). Based on molecular data, *S. simulans* is usually isolated from milk with mastitis, but *S. chromogenes* can be associated with subclinical mastitis and skin microbiota (24, 100). *S. epidermidis* colonizes the teat apices of dairy cows and healthy human skin (108, 109). NAS inhabit different ecological niches, including bedding materials and different parts of the animal body, including udder and teat skin, nostrils, and teat canal (110). The epidemiological distribution of these groups of bacteria, their spread mechanisms, and reservoirs vary and are affected by environmental, managemental, and host factors (19, 22, 111, 112). The natural habitat of each species needs to be determined to differentiate environmental and host-adapted species (64, 113) to design appropriate control measures for these groups of bacteria.

#### 2.2.2 Genetic diversity and virulence factors of NAS

NAS are genetically different in their ability to cause mastitis in dairy cows (114, 115). They have species-specific virulence factors and pathogenicity that affect the productivity of dairy animals. NAS also form a biofilm that enables them to colonize milking utensils and milkers’ hands, which helps their spread and transmission (116, 117). They also vary in their susceptibility to antimicrobial drugs (19, 112).

#### 2.2.3 Host immune responses against NAS IMI

Macrophages are the first responders of the innate immunity in the mammary glands, with the subsequent recruitment of neutrophils from systemic circulation into the mammary glands (118). *Staphylococcus* species vary in their ability to induce inflammatory reactions in the mammary glands and increase SCC, with the highest counts usually caused by *S. aureus.* However, NAS, such as *S. chromogenes*, *S. hyicus*, *S. agnetis*, *S. simulans*, and *S. xylosus* are also reported to cause increased SCC similar to *S. aureus* (87, 119). Staphylococcal IMI, especially *S. aureus,* usually increases SCC initially, which leads to subclinical mastitis. If *S. aureus* resists clearance by host defense, the infection becomes chronic, and SCC decreases to a modest level (120). NAS occasionally causes clinical mastitis with SCC, usually ranging in the low to moderate increase, but may cause significantly increased SCC (22).

In experimental challenge infection, *S. simulans* caused more inflammatory reactions than *S. epidermidis* (121). Similarly, in field studies, *S. simulans* caused more clinical mastitis than other NAS (101, 106). Another study found that *S. chromogenes* originally isolated from milk with mastitis induced more inflammatory reactions than *S. chromogenes* originated from teat apex (122). However, it is unclear if this difference is because strain differences in virulence or teat skin colonizing strains are non-pathogenic microbiota. In contrast, strains from intramammary areas are pathogenic microbiota. In another study, *S. epidermidis* and *S. haemolyticus* were shown to cause high SCC (123). In some studies, a slight increase above 100,000 cells/mL was reported for quarters infected with NAS (109, 124), whereas in another study, SCC varied from as low as 70,000 cells/mL to as high as 123,000 cells/mL of milk depending on the species of NAS involved (20). Some NAS species, such as *S. agnetis*, *S. hyicus,* and *S. simulans,* cause clinical mastitis more frequently than others (101, 104, 105), whereas some others, such as *S. epidermidis* cause mild inflammatory responses compared to *S. simulans* (121). However, *S. epidermidis* was also reported to cause high SCC in subclinical cases of mastitis (123).

Another study found that *S. agnetis* was more phagocytosed by murine macrophages than *S. simulans* or *S. chromogenes* but more resistant to killing by phagocytic cells similar to *S. simulans* and *S. aureus,* whereas *S. chromogenes* was more efficiently killed than *S. simulans* and *S. agnetis* (125). Despite observed differences in opsonophagocytic killing of *S. simulans* and *S. chromogenes* by phagocytic cells, both can exist in the mammary glands throughout lactation with increased SCC (103, 126). In another study, *S. haemolyticus* was better phagocytosed by blood neutrophils than *S. aureus* and *S. chromogenes,* and both *S. aureus* and NAS did not prevent intracellular reactive oxygen species (ROS) production in blood and milk neutrophils (127). The authors showed that *S. chromogenes* induced less ROS in milk neutrophils than *S. aureus* but induced ROS comparable to *S. aureus* from blood neutrophils and more ROS from blood neutrophils than *S. haemolyticus.* Transcripts and protein level evaluations of expression of proinflammatory chemokines and cytokines in the udder of cows with chronic mastitis due to coagulase-positive and coagulase-negative Staphylococci showed no difference between the Staphylococci (128). In another study, *S. aureus* was known to cause persistent intramammary infection-induced proliferation of CD4+ and CD8+ lymphocytes, whereas *S. aureus* (originated from nostrils) and *S. chromogenes* strains (known to cause persistent IMI) had no effect on T and B cell proliferation (129). The authors showed that both *S. aureus* and *S. chromogenes* originating from persistent IMI significantly increased IL-17A and IFN-γ production from peripheral blood mononuclear cells. Peripheral blood mononuclear cells (PBMC) from multiparous cows produced significantly higher IL-17A and IFN-γ; multiparous cows tend to have a higher B-lymphocyte and a lower T-lymphocytes proliferative response than primiparous and nulliparous cows.

Staphylococci can resist opsonophagocytic killing by forming capsules and other extracellular polysaccharides (130–132). There are differences among NAS species in their susceptibility to opsonophagocytic killing by macrophages (125). The pathogenic mechanisms responsible for the differences between NAS and *S. aureus* strains are still unknown and need further investigation. These differences could be due to yet unknown novel virulence factors. Therefore, further investigation is required.

#### 2.2.4 Role of NAS on udder health, milk quality, and SCC

The prevalence of NAS in quarter milk samples in the US and European dairy cattle farms ranges from 27 to 55% (21, 133). Similarly, the prevalence of NAS in bulk tank milk of herds ranges from 43% to 60 or 90% (7, 19, 134). In different countries, NAS species are increasingly reported as an etiology of subclinical mastitis in cows, goats, and sheep (135). Differences in cattle housing, grouping, and age affect NAS prevalence and bacterial count (64). Variations in study methodologies and methods of species identification affect the prevalence assessment of mastitis due to these groups of bacteria (113, 136).

Some studies consider NAS as minor pathogens that cause only a slight increase in SCC and mild clinical mastitis (CM) with no effect (137–139) or little effect on milk production (96, 101, 124, 140–146) In contrast, others report a higher milk production in infected animals than in noninfected animals (142, 147). Some investigators reported no differences among NAS species in individual quarter milk SCC (9, 148), while others reported differences between species (119, 149). A recent study reported that IMI with *S. chromogenes* early in lactation led to a significantly increased quarter SCC (124). Some NAS species, such as *S. chromogenes*, *S. simulans*, and *S. xylosus,* induced increased SCC comparable to *S. aureus* (119, 149). Similar to differences observed for the effect on SCC, species-specific differences in persistence were also reported (19, 103, 119, 123). NAS can cause increased SCC (142) and play a role in clinical mastitis development in well-managed herds (142).

The persistence of NAS IMI depends on the specificity of the species involved. Persistent IMI by *S. chromogenes* and other NAS species induce increased SCC compared to transiently infected quarters (145). However, the authors concluded that both transient and persistent IMI were not significantly associated with quarter milk yield during early lactation (145). Yet, milk yield from quarters recovered from *S. chromogenes* IMI was significantly lower than uninfected quarters (145), which might indicate some sequential effect in milk production.

NAS species induce only mild inflammatory response with mild to moderate increase in SCC in the infected quarter, reducing milk quality and price, and low bulk tank milk SCC may discourage producers from intervening in IMI, allowing these pathogens to cause continuous loss of productivity (124, 143, 146). In dairy cows with subclinical infection with these groups of pathogens at peak lactation can result in approximately 1.8 kg/d reduction in milk production (94, 146). Because of a modest increase in milk SCC, the IMI due to NAS may not account for increased SCC in dairy farms that already have high SCC due to major mastitis pathogens. Data from farms also showed that NAS species are more prevalent in farms with low bulk tank milk SCC (8, 142), which may indicate that current mastitis control measures that reduce the incidence of some contagious bacteria such as *S. aureus* and *S. agalactiae* may not be effective on NAS. The occurrence of mastitis due to these groups of bacteria varies with farms, and economic losses due to subclinical mastitis of these bacteria are difficult to estimate due to the absence of easy and producer-friendly accurate diagnostic tools at the farm level (146, 147, 150). Similar to differences observed for the effect on milk SCC, species-specific differences in persistence have also been reported (19, 103, 119, 123). All these observations clearly indicate that further detailed investigations at the individual species level are required to determine the role of each species in bovine mastitis. Therefore, it is important to study each species of NAS individually and determine their virulence factors, pathogenicity to the host, and disease pathogenesis mechanisms to determine their role in causing mastitis, milk quality, and economic losses.

Some NAS species produce different antimicrobial agents, including bacteriocin, subtilosin A, lysostaphin, and Lugdunin, potentially protecting the colonization of udder or their microenvironmental niches by other bacteria (151–154). Under *in vitro* conditions, NAS species inhibit biofilm formation by bacterial mastitis pathogens (155), and metabolites from NAS species prevent the expression of *S. aureus* agr-related genes known to regulate the expression of virulence genes (156). Similarly, under *in vivo* conditions, the udder, pre-colonized by some strains of NAS, was shown to resist colonization by major bacterial mastitis pathogens (157–160). However, even though pre-colonization of the udder by some members of NAS species seems protective against colonization by major mastitis pathogens, some NAS species themselves were isolated and identified as the etiology of mastitis and shown to be responsible for milk production losses (94, 142, 146). It has also been shown that priming the murine mammary glands with *S. chromogenes* induced innate responses that reduced the growth of *S. uberis* (161). However, the authors did not clearly demonstrate if priming with *S. chromogenes* itself induced mastitis rather than enhancing protective innate immunity. Another study showed that intramammary challenge with *S. chromogenes* during a dry period resulted in colonization of challenged quarters by *S. chromogenes,* which induced high SCC, IFN-γ, and IgG2 production in challenged quarters but lower IL-6 and IL-10 in both challenged and colonized and non-colonized quarters (162). To conclude these findings as protective, it is important to determine how long the colonized quarters were shedding *S. chromogenes* without causing mastitis and if intramammary infusion of other bacterial mastitis pathogens into these *S. chromogenes* colonized quarters can prevent IMI or mastitis. Detailed controlled experimental challenge studies under *in vivo* conditions in dairy cows are critically needed to determine the roles of colonization of udder quarters by specific NAS species on mastitis status, milk quality, and milk production losses.

#### 2.2.5 Therapeutic measures and antimicrobial resistance of NAS

Staphylococci are known to become resistant to several antibiotics, including methicillin resistance, which is important for public health (163, 164). Methicillin-resistant *Staphylococcus aureus* (MRSA) infection can only be treated with limited antibiotics and needs long-term treatment (163, 165–167). MRSA infection is zoonotic (168), and continuous antimicrobial susceptibility surveillance is very crucial to control the transmission of this strain from animal production to humans and vice versa (169). They may transfer resistance traits to *S. aureus* or other bacteria, resulting in the emergence of multidrug-resistant strains (94, 135). The prevalence of infection by these groups of bacteria is on the rise mainly due to the spread of drug resistance among these groups (135). The most frequently seen resistance among staphylococci is resistance due to the production of β-lactamases, with more common production among subclinical non-aureus staphylococci isolates than clinical isolates (170). They exhibit resistance to multiple classes of antimicrobial drugs (32, 171, 172). The response to the treatment of *S. aureus* mastitis during lactation is poor (30, 173–175), with a 25–75% quarter cure rate for treatment at dry-off and 3–63% for short-term treatment during lactation (174, 175).

A recent antimicrobial susceptibility study involving *S. aureus* and NAS from bovine mastitic milk samples in Finland showed the presence of the *blaZ* gene and penicillin resistance of 9.3% in *S. aureus* and 28.9% in all NAS (176). The proportion of penicillin-resistant isolates was highest in *S. epidermidis* and lowest in *S. simulans*. The *S. epidermidis* is the predominant species carrying the *mecA* gene. Some phenotypically penicillin-susceptible staphylococci have the *blaZ* gene, but isolates negative for *blaZ* or *mec* rarely manifest resistance, indicating that genotypic AMR testing (176) may be good for the choice of antimicrobial drug for treatment. Another study from Switzerland to determine intramammary microbiome and resistome from the milk of healthy dairy cows reported a high prevalence of resistance to clindamycin and oxacillin (65 and 30%, respectively) in *S. xylosus* but not associated with chromosomal or plasmid-borne ARGs (177). The authors found that most resistance was justified by the presence of mobile genetic elements such as *tetK*-positive plasmids.

### 2.3 Universal staphylococcal virulence regulators

**Figure 1:**
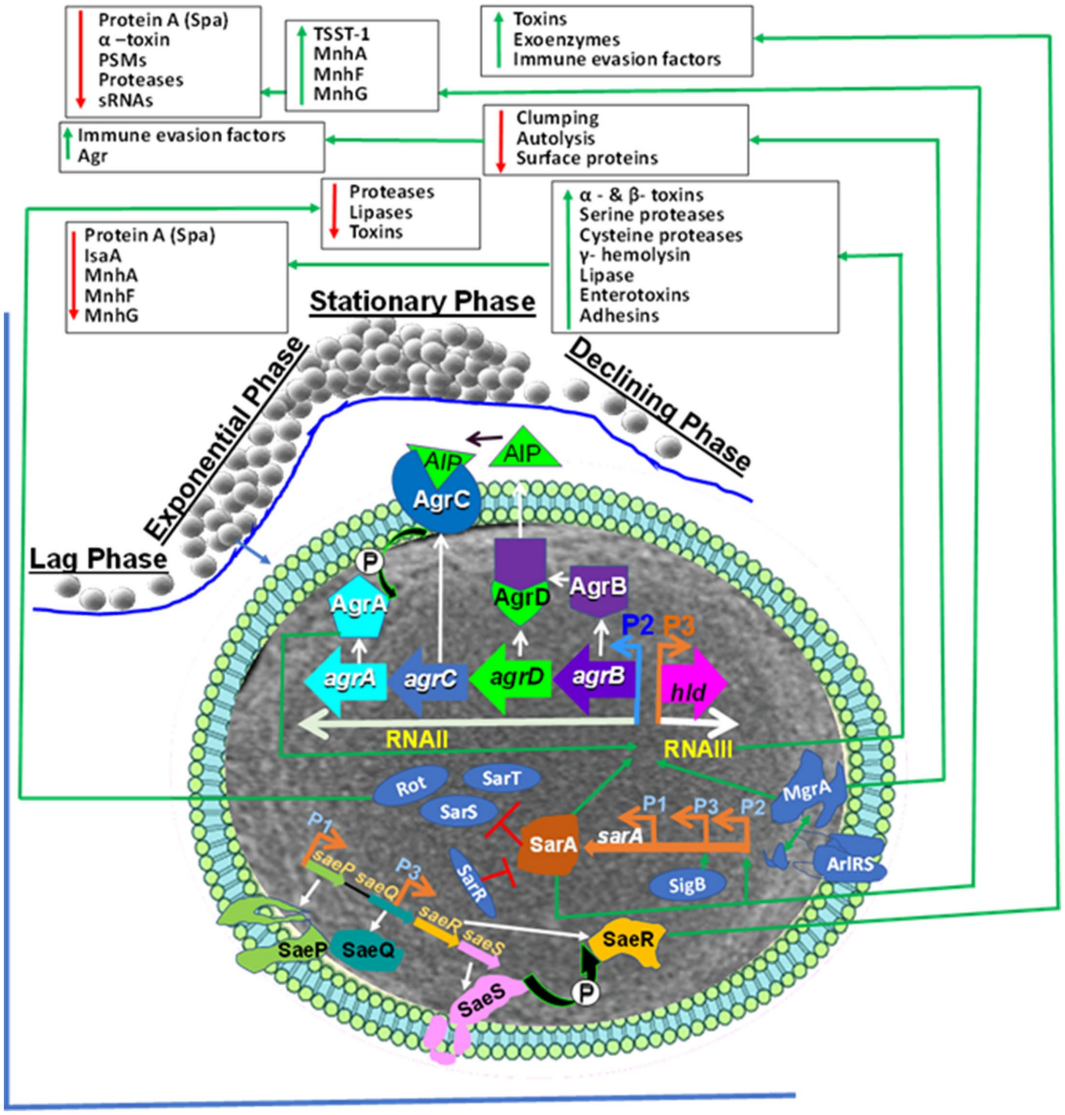
Staphylococcus aureus Universal Virulence Regulators. AgrA: accessory gene regulator A; AIP: Autoinducing peptide (AIP); SarA: staphylococcal accessory regulator A; SaeRS: S. aureus exoprotein expression locus RS; AgrAC: accessory gene regulator AC AgrAC; Rot: repressor of toxin; SigB: the alternative sigma factor; ArlRS: the autolysis regulated locus RS., Spa: Staphylococcal protein A, psms: phenol-soluble modulins, sRNAs: Small RNA regulators, TSST-1: toxic shock syndrome toxin-1. The S. aureus global regulators consist of the agr, ArlRS, SaeRS, and the SarA homologs (SarA, Rot and MgrA). The agr system induction causes expression of toxins and enzymes.The AIP is encoded from AgrD. The AgrD is processed to AgrB by SpsB peptidase. The extracellular AIP is detected by histidine kinase AgrC. This induced phosphorylation that transfers phosphate to AgrA that induces activation and binding to the P2 and P3 promoters inducing expression of RNAII and RNAIII, respectively. The RNAII comprises agrBDCA operon that encodes AgrB, AgrD, AgrC and AgrA. RNAIII is the major effector of the agr system through inducing target genes. Activated AgrA binds to promoters of PSMs genes and induces their expression. The SaeRS induce expression of exo-proteins. The SaeS phosphorylates its associated response regulator SaeR. This cause activation of SaeR which binds to the promoter region and induce expression of different virulence factors. The sae gene consists of saeP, saeQ, saeR and saeS that are under the control of the P1 promoter. SarA: sarA is induced from P1, P2 and P3 promoters and trigger expression of exo-proteins but represses spa. The alternative sigma factor σB (SigB) induces sarA through binding to the P3 promoter and prevents agr activity. The SarR binding to all three promoters prevents expression. SarA is an inducer of the agr system, and it represses the three SarA-like proteins SarH1, SarT and Rot. Rot regulates toxins and extracellular proteases and agr activation prevents Rot translation. MgrA: Induces expression of efflux pumps and capsule but represses surface proteins. The ArlRS induced by unknown factor and then activate MgrA but represses agr and autolysis. It down-regulates surface proteins, enabling ClfA/ClfB to interact with fibrinogen.

Staphylococci are opportunistic commensal bacteria (23, 178) that can cause different diseases such as superficial skin infections, endocarditis, osteomyelitis, necrotizing fasciitis in humans (179) and mastitis, necrotizing endometritis, pyometra, exudative epidermitis, cystitis, and otitis in animals (30, 180). However, it is important to emphasize that the virulence factors of human-adapted and bovine-adapted strains may differ. Nevertheless, understanding similarities and differences between bovine-adapted strains and human-adapted strains at cellular and molecular (genomic, transcriptomic, proteomic, and metabolomic) levels is critically important to control infection caused by *Staphylococcus*. To inhabit or colonize different hostile microenvironmental niches, such as in the host body, *S. aureus* regulates the expression of its different virulence genes (181). The function of these different virulence factors can be attachment to host cells, immune evasion, nutrient breakdown, and acquisition (182, 183). The virulence factors of *S. aureus* and NAS are encoded from the chromosome and mobile genetic elements [e.g., phages or prophages, plasmids, pathogenicity islands (SaPIs), and staphylococcal cassette chromosome mec (SCCmec)] (182, 184). These different pathogenicity factors are controlled by universal virulence regulators (regulons), such as the two-component regulatory systems (TCS) that comprise 16 different TCS (185–187), and the DNA binding cytoplasmic proteins, such as the staphylococcal accessory regulator A (SarA) (188). Its homologs SarR, SarS, SarT, and other protein families (189–191) are essential for the pathogenesis of *S. aureus* infections. The TCS, such as the accessory gene regulator AC (AgrAC) (187), the *S. aureus* exoprotein expression locus RS (SaeRS) (192, 193), the staphylococcal respiratory regulator AB (SrrAB) (194–196), and the autolysis-regulated locus RS (ArlRS) (197–199) regulate the expression of many virulence factors at different growth phases of the staphylococci (Figure 1). Out of the 16 TCS, the WalKR (WalK-histidine kinase and WalR-response regulator) controls cell wall metabolism and is essential for the viability of *S. aureus*; the other 15 are not active in multiple strains (200–202). *S. aureus* survives in the hostile host body or environmental niches by coordinated expression of its cytoplasmic regulators (185). These include the SarA family of regulators, repressor of toxin (Rot), multiple gene regular A (MgrA) (203), alternative sigma factors (SigB and SigH), and various TCS such as AgrCA, SaeRS, SrrAB, and ArlRS.

SarA and SaeRS act together to decrease protease production and help in biofilm formation in *S. aureus* (204). The *sarA* mutation decreases biofilm but increases sensitivity to antibiotics and the expression of alpha toxin, which is a pathogenicity factor. *saeRS* induces the transcription of *fnbA* and other *S. aureus* surface proteins. The *saeRS* mutation decreases surface proteins and biofilm formation (204) but increases efficacy with antimicrobial treatment (205). The mutation of *sarA* increases extracellular proteases, which decrease the ability to bind to fibronectin, therefore limiting the accumulation of surface-associated proteins. Several of these regulatory mechanisms have not been well studied in bovine-adapted strains of staphylococci. It is very important to understand the regulatory mechanisms of both human-adapted and bovine-adapted strains, their similarities and differences, and how these regulatory mechanisms change if human-adapted strains infect bovine or vice versa.

#### 2.3.1 Staphylococcal virulence factors

A study on the presence of a total of 296 virulence factors in *S. aureus* from bovine mastitis found 219 were present in all isolates (85). The authors concluded that there was no association between the presence of virulence factors and clinical outcomes of infection, but the presence of virulence factors varied with clonal complexes.

The presence of virulence genes and antimicrobial resistance genes varies among *S. aureus* isolates from bovine mastitis. The major factor causing disease is not the presence or absence of a specific virulence factor or resistance gene in a given isolate. Instead, it is their opportunistic pathogenic ability to acquire any virulence gene or resistance gene under certain environmental pressure. However, the ability to acquire mobile genetic elements that may disseminate within or across different lineages is much more important (206). It has been shown that the SOS responses from antimicrobial drug pressure promote horizontal gene transfer of pathogenicity islands (207, 208).

*S. aureus* has different virulence factors (VFs) that are responsible for mastitis pathogenesis, such as adhesion and internalization into host cells, tissue damage, evasion of host immunity, and getting nutrients from the host (209, 210). However, detailed pathogenic mechanisms and effects of several VFs in mastitis pathogenesis are still poorly defined. The disease severity is influenced by the expression of virulence genes (211) of the pathogen, the immunological defense of the host, and environmental stress factors (212). However, understanding detailed mechanisms of pathogenesis and associated symptoms needs further investigation (213).

A comparative analysis of *S. aureus* and NAS virulence factors from clinical and subclinical bovine mastitis did not show any association between the presence of any virulence factors and the clinical outcome of mastitis (214). Similarly, a comparative genomic analysis of *S. aureus* from subclinical and clinical bovine mastitis did not find any association between the presence of virulence genes and the clinical outcome of mastitis (215). However, the authors found that *S. aureus* from clinical and subclinical mastitis were separated based on sequence variation of membrane-bound lipoprotein (215). However, another genomic study on *S. aureus* from clinical and subclinical mastitis reported an association of multiple genes with the clinical outcome of mastitis (216), but these genes were clustered in the same clonal complex (CC). Some authors suggest that a combination of certain virulence genes appears to cause mastitis than any single virulence gene (213). One study reported some level of differences in the virulence genes of *S. aureus* isolates from subclinical and gangrenous mastitis in sheep (217).

A study on the presence of known virulence genes and their regulation in *S. aureus* isolates from bovine mastitis found that all isolates were in Agr I and II classes, but sarT and *sarU* were lacking in some isolates. On the other hand, *sarB* and *sarD* were absent from all isolates. Most of the regulatory genes were present in all bovine isolates. The rot gene coding for the transcriptional regulator was present in all bovine isolates (85). The authors reported that toxins were variably present in *S. aureus* from bovine mastitis (85). Another study reported the presence of all hemolysin genes in *S. aureus* from bovine mastitis (85), and all were negative for the chemotaxis inhibitory protein of *S. aureus* (CHIPS) but were positive for the staphylococcal complement inhibitor gene (scn) (85). It has been shown that the presence of genes coding for cell-wall-anchored proteins such as *sasC*, *sasD*, *sasF*, *sash*, *sasG,* and *sasK* varies among bovine isolates but *sasB* and *bap* gene was absent from all isolates (85, 218).

Intracellular invasion and infection were possibly mediated by the cysteine proteases SspB and SspC, which were evident in all the isolates (219). Proteins associated with bovine immune invasions, such as Sbi, Cap, and AdsA, were identified in the isolates. All the isolates demonstrated crucial virulence characteristics, including hemolysis induction and biofilm formation (220). Some isolates were positive for *agr* and *sarA* systems associated with quorum sensing (221). All isolates were positive for intercellular adhesion, such as *icaA*, *icaB*, *icaC*, *icaD*, and *icaR* (220). Some isolates were positive for the spa gene (222). All isolates were positive for Ssp serine protease, which is responsible for *in vivo* multiplication and intracellular survival (223). The majority of the isolates were positive for the second immunoglobulin-binding protein (Sbi), which is responsible for immune evasion (224). All the isolates were positive for serotype eight capsular polysaccharide (Cap) and adenosine synthase A (AdsA), which are responsible for bovine immune evasion. All isolates were also positive for cysteine proteases (staphopain B [SspB] and staphopain C [SspC]), which enable biofilm production and intracellular colonization of *S. aureus* (219, 225).

*Staphylococcus aureus* and NAS virulence factors can be divided into two groups: (1) non-secretory or cell wall-associated structural parts and (2) secretory parts.

#### 2.3.2 Non-secretory virulence factors

These are surface proteins associated with the peptidoglycan cell wall that help to colonize host tissues (226) during staphylococcal pathogenesis. Additionally, non-secretory surface proteins are involved in evading host immune responses, invading host cells and tissues, and forming physical barriers such as biofilms.

**Staphylococcal protein A (SpA)** is present in the cell walls of *S. aureus* and NAS. It binds Fcγ domains of the IgG and prevents the immunoglobulin-mediated removal of *S. aureus* from the body (227). It also binds the Fab of IgM cross-linking B-cell receptors, which leads to the programmed death of B lymphocytes (228). Consequently, immunoglobulins cannot effectively clear *S. aureus* infection due to the effects of protein A (229).

##### 2.3.2.1 Biofilm formation

A biofilm is an extracellular matrix composed of exopolysaccharides, surface proteins, and nucleic acids (230, 231) that protect bacteria against host immunity and antimicrobial drugs (232–234). Biofilms bind to the host tissue surfaces by polysaccharide intercellular adhesin (235). Proteases promote the detachment of attached bacteria and increase entry into intracellular areas or invasion (236). The biofilm formation by *S. aureus* may enhance their colonization of the mammary gland and protection from host phagocytic cells (237, 238), resulting in chronic mastitis (239–242). However, the role of biofilm formation in mastitis pathogenesis remains unresolved and needs detailed *in vivo* study.

A previous study on 90 NAS found that barring a few (3.3%), the majority (96.7%) of them had some ability to form a biofilm (243). Other studies also found that 90% of NAS were positive for biofilm, and at least 11 species were identified in each study (244–247).

Staphylococci form biofilm through different mechanisms (235) that vary with species and the microenvironmental niche (237). Some of the mechanisms include the production of polysaccharide intercellular adhesin (PIA), surface proteins including biofilm-associated protein (Bap) (230, 248), slime, teichoic acids, and extracellular DNA (eDNA) (249–251).

The intercellular adhesin (*ica*) operon encodes different proteins (IcaA, IcaB, IcaC, IcaD, and IcaR) (235, 252, 253). Each of these proteins has a different function; for example, IcaR controls the *ica* operon, the induction of *icaA* and *icaD* at the same time promotes slime formation, and *icaC* encodes receptor protein (249, 250, 254). The presence or absence of these different *ica* genes in this operon also varies with strains. A previous study found that approximately 24.1 and 21.4% of NAS isolates were positive for the *icaA* and *icaD* genes, respectively (255), whereas all *S. aureus* isolates (100%) were positive for the *icaD* gene (255). The majority (73.2%) of NAS were positive for *icaA* and *icaD* genes (256). However, the majority (81.7%) of the *icaA* and *icaD* positive NAS were negative for the *bap* gene (256). Contrary to *S. aureus*, despite being negative for *icaA* and *icaD* genes, NAS species form a biofilm, indicating that these genes are not always essential for phenotypic mechanisms (256).

Slime is an exopolysaccharide layer or extracapsular layer of some biofilm that increases adhesion to host cells and protects bacteria from opsonophagocytic killing and the effect of antibiotics but is not found on all biofilms (257, 258). The formation of biofilm/slime depends on the strain. A study on staphylococci reported that 80% of *S. aureus* produced slime and formed strong biofilms (255), whereas approximately 87 and 84.2% of NAS with and without slime formation, respectively, produced strong biofilms (255).

Biofilm-associated protein is a high-molecular-weight surface protein responsible for cellular aggregation and biofilm formation in staphylococci (259, 260). *Staphylococcus aureus* from cases of bovine mastitis may carry *ica* and bap genes, be positive for the *ica* gene but negative for the *bap* gene, or be negative for both (261). A previous study (261) showed that *bap*-positive *S. aureus* was more able to cause IMI and less susceptible to antibiotics if it produced biofilm *in vitro*, which may show the enhancing ability of Bap and associated chronic *S. aureus* IMI.

An evaluation of the link between the presence of *ica* locus genes, slime formation, and the presence of Bap protein with biofilm formation did not show a consistent association of biofilm formation with any of these factors. A study on *S. aureus* from cases of bovine mastitis showed that all isolates tested carry *icaA* and *icaD* genes (262, 263), most of which were slime producers (262). The presence of *bap, icaA,* and *icaD* was linked with biofilm synthesis. However, most *S. aureus* isolates negative for these genes were biofilm formers (264). Similarly, all slime-positive ones could not form biofilm *in vitro* (262). Therefore, the presence of *ica* genes is linked with biofilm; however, *ica* genes are not mandatory for biofilm production since some *ica*-negative *S. aureus* can produce biofilm using different mechanisms (265, 266).

##### 2.3.2.2 Role of biofilms in the pathogenesis of bovine mastitis

The role of biofilms in bovine mastitis is still unclear. Most studies on the role of biofilm in bovine mastitis were focused on the characterization of the biofilm-forming capability of different bacterial mastitis pathogens *in vitro* using different methods (microtiter plates with crystal violet staining for bacterial biomass quantification, Congo red Agar test, and standard tube method for biofilm formation assay) (116). The majority of *S. aureus* isolates from cases of mastitis form biofilm *in vitro*, but that may not be the case under *in vivo* conditions. The physiological characteristics of biofilm formation *in vitro* are different from *in vivo*, as also seen with *P. aeruginosa* during human infections (267). The role of biofilm in human infections is well known since the finding of bacterial aggregates in the lungs of cystic fibrosis patients (268) in 1977 and the first report of a medical biofilm causing recurrent infection in 1982 (269). Despite these findings in human medicine, most studies focus on *in vitro* characterization in veterinary medicine. In human medicine, biofilm is responsible for several diseases ranging from wound infections to lung infections, osteomyelitis, urinary tract infections, dental plaque, and endocarditis (270).

*In vivo*, there are interactions among bacteria, host immune response, and antimicrobial drugs administered for treatment, which is not the case under *in vitro* conditions. Therefore, more *in vivo* studies on dairy cows are required to determine the role of biofilm in the pathogenesis of *S. aureus* and NAS mastitis. Only two studies have reported biofilm formation inside the mammary glands of dairy cows with mastitis (271, 272). One reported the clustering of *S. aureus* bacteria in the alveolar lumen and lactiferous ducts of mammary glands of experimentally challenged cows using microscopy (271). The second study reported the presence of polysaccharide intercellular adhesions (PIA) in the swabs obtained from different parts of the mammary glands of slaughtered dairy cows with *S. aureus* mastitis using fluorescence microscopy (272). One study found that *S. aureus* biofilm had less invasive ability in mammary epithelial cells compared to planktonic *S. aureus* cultures, and the biofilm culture triggered less cellular response than the planktonic cultures. Both planktonic and biofilm forms of culture triggered the induction of IL-6 by mammary alveolar cells, which could be an anti-inflammatory response (273). This is in line with the role of biofilm in human disease, where biofilms do not induce any specific immune responses (274) when the cell density is low to avoid detection by immunity but increase expression of the virulence factors (275) when cell density is high. However, *in vitro* studies showed no difference in host cell invasion between biofilm former and non-biofilm former (276, 277). The most important question is how biofilm resists host immunological responses (278). More detailed *in vivo* studies in dairy cows are needed to determine the role of biofilm in the pathogenesis of bovine mastitis. Currently, the most preferred diagnostic method to detect bacterial biofilms in tissue is peptide nucleic acid fluorescence *in situ* hybridization (PNA-FISH), which uses probes that hybridize to bacterial ribosomal RNA that can be detected by confocal laser scanning microscopy (CLSM). This is a sensitive method preferred in the research on biofilm in humans (279–283). This method can be used on mammary glands in dairy cows.

Detailed knowledge of the genotypic and phenotypic requirements of *S. aureus* and NAS to produce biofilm, especially *in vivo*, may improve our understanding of the pathogenesis of staphylococcal IMI and may allow us to develop methods to disintegrate or decrease biofilm formation or increase its removal.

##### 2.3.2.3 Coagulase, von Willebrand factor binding protein, and staphylokinase

These staphylococcal proteins serve as cofactors to activate host zymogens (284). Coagulase (Coa) and von Willebrand factor binding protein (vWbp) interact with prothrombin, causing activation of zymogen (inactive form) that converts fibrinogen, a plasma protein produced by the liver, to fibrin. Fibrin catalyzes blood clot formation, inhibiting bacterial killing by phagocytic cells (284–286). Staphylokinase (Sak) is encoded from lysogenic phage and interacts with plasmin in serum, leading to the conversion of plasminogen to plasmin, resulting in the lysis of fibrin clots (287).

#### 2.3.3 Staphylococcal secretory (secreted) virulence factors

**Exotoxins** are secreted toxins that represent approximately 10% of the total secretory product of *S. aureus* (288). The majority of *S. aureus* isolates from cases of bovine mastitis produce exotoxins such as hemolysins, nucleases, proteases, lipases, hyaluronidase, and collagenase (289). Staphylococcal exotoxins can be divided into cytotoxins and superantigens. Cytotoxins damage host cell membranes, causing target cells lysis and inflammation. Superantigens induce increased cytokine production and trigger B and T cell proliferation.

##### 2.3.3.1 Cytotoxins or cell membrane-damaging toxins

**Staphylococcal α-toxin** (hemolysin-α or Hla) is a 33 kDa pore-forming toxin encoded by the *hla* gene from chromosome through *agr* system and causes membrane damage and cell lysis (290, 291). It causes the lysis of different cells (e.g., erythrocytes, platelets, endothelial cells, epithelial cells, and certain leukocytes) (292, 293). It binds to A Disintegrin and metalloproteinase domain-containing protein 10 (ADAM-10) receptors on cells that determine its species and cell type specificity (294). In mice, it causes cleavage of E-Cadherin, which is the junction protein, and the loss of the epithelial barrier (295). **β-toxin** (hemolysin-β or Hlb) is non-pore forming but causes hydrolysis of the sphingomyelin component of the cell membrane (leukocytes and red blood cells) (296). **γ- toxin** (hemolysin-γ or Hlg) is a bi-component (S [slow, HlgA or HlgC and F fast, HlgB]) pore-forming toxin encoded from core genome where F binds to phosphatidylcholine of cells, and S binds to cell membranes causing lysis (macrophages, neutrophils) and monocytes (297, 298). **δ-toxin** (hemolysin-δ or Hld) causes lysis of neutrophils, monocytes, and degranulation of mast cells (299). All (α, β, and γ) toxins require specific receptors, but δ-toxin does not require a specific receptor to cause cell lysis and is believed to belong to phenol soluble modulins (300).

**Phenol soluble modulins** (**PSM**) are amphipathic (both lipophilic and hydrophilic) peptides encoded from psmα and psmβ operons on the chromosome and induced by the *agr* system (298). The PSM causes cell death, biofilm production, and modulation of immunity (284). α- and β- hemolysins and PSM induce breaks in the cell membranes of the immune cells and trigger inflammatory reactions (301). A previous study showed that approximately 69% of hemolytic *S. aureus* isolates are positive for β-toxin, which may indicate its effect on virulence and pathogenicity (233). The α- and β- hemolysins enhance invasion and exacerbate the spread and transmission of infection (233). The ability to invade cells and stay in the intracellular area enhances chronic recurrent infection (235).

**Leukocidins** are 32–35 kDa toxins encoded on the core genome or phage (298) that cause damage to leukocytes such as macrophages, neutrophils, monocytes, and dendritic cells (227, 236, 302). LukMF is encoded by the temperate phage ΦSa1 and is present in most *S. aureus* isolates of bovine, ovine, and caprine mastitis cases (303, 304). It binds to the C-C chemokine receptors, also known as beta-chemokine receptors (CCR1, CCR2, and CCR5) on neutrophils and macrophages, leading to cell lysis (293, 305).

##### 2.3.3.2 Staphylococcal superantigens

Staphylococcal superantigens bind to T cell receptor (TCR) Vβ domains on T cells with major histocompatibility complex (MHC) class II protein on antigen-presenting cells (APC) that result in activation and proliferation of T cells without antigen processing and presentation (299). T-cell superantigens are exotoxins produced by *S. aureus* that range between 19–30 kDa and are resistant to heat, proteolysis, and desiccation (306). There are also superantigen-like proteins, previously called staphylococcal enterotoxin-like proteins (307). However, because of their lack of emetic but strong mitogenic properties, they were renamed staphylococcal superantigens (308). They are mainly involved in immune evasion (309). They are broadly divided into staphylococcal enterotoxins (SEs), Staphylococcal enterotoxin-like superantigens (SE-ls), and toxic shock syndrome toxin-1 (TSST-1).

**Enterotoxins** are water-soluble, stable extracellular proteins that are resistant to heat and enzymatic degradation (310–312). Enterotoxins include SEA, SEB, SECn, SED, SEE, and SEG (313) that bind to receptors on the host cell surface and trigger a series of signaling and responses inside the cell, causing emesis (308). They are superantigens that bind to MHC-II outside antigen binding site and to T-cell receptors on CD4+ cells and trigger potent polyclonal activation of T cells and increased release of inflammation mediating cytokines that lead to shock and death (236).

The presence of enterotoxin genes and the protein production capability of NAS species are still being studied, and there is a lack of understanding of their enterotoxigenic effects (314). NAS from bovine IMI tends to have variable SE genes that are continuously being lost with proceeding generations compared to *S. aureus* isolates containing SE genes (315, 316).

Staphylococcal food poisoning is intoxication due to the consumption of food that contains preformed enterotoxins from staphylococci that multiply in food that is inappropriately stored or handled (317–319). The first staphylococcal food poisoning was reported in 1884 in Michigan (US) by Vaughan and Sternberg due to the ingestion of contaminated cheese (320). Staphylococcal enterotoxins are produced over different temperatures, pH, salt concentrations, and water content (321). *S. aureus* can be killed by heating the food, but the SE remains active and can cause food poisoning (310). *Staphylococcus aureus* grows well in milk and milk products, which is a main source of human infection (322).

Two major factors for *S. aureus* multiplication and growth are improper milk storage temperature and unhygienic handling of foodstuff (322, 323). Higher starch and protein in food, pH, water activity, and warm temperature increase enterotoxin production (322). *S. aureus* can survive in a pH of 4.5–7.0, a low water activity of 0.86, and a salt concentration of up to 20%, which would normally kill bacteria (324, 325). Lower pH decreases *S. aureus* attachment to solid surfaces, subsequently decreasing the ability to colonize and cause infection (326).

The *sea* gene is present in temperate bacteriophages, and when bacteriophages infect bacteria, it becomes integrated into the bacterial chromosome as a prophage and remains as part of the genome (327). Under stressful conditions of improper food preservation, the prophage gets activated, multiplies the phage genome, and produces new bacteriophages (328). To avoid the multiplication of *S. aureus*, milk must be refrigerated at all times, from production to consumption (310, 329). Milk should be pasteurized to kill pathogenic bacteria in milk, but pasteurization does not detoxify already produced enterotoxins (330, 331).

Milk from cows with subclinical mastitis due to NAS, if consumed, can affect human health in different ways (113, 150). Therefore, the consumption of raw milk must be discouraged, and pasteurization of milk is recommended for safety and improved shelf life (146). Even though proper pasteurization is expected to kill pathogenic bacteria, the mobile genetic elements (e.g., plasmids) mediated resistance genes in bacteria may not be destroyed by pasteurization and could transform the carrier bacteria to become viable but nonculturable (VBNC) form (332, 333). Toxins produced by NAS due to inappropriate cooling during manufacturing and post-processing contamination are resistant to extreme heating or cold and can cause foodborne intoxication (146, 150).

The roles of different virulence factors of *S. aureus* and NAS in the pathogenesis of mastitis in dairy cows require detailed study since most of *S. aureus* and NAS isolates from cases of bovine mastitis are known to carry these virulence genes, but their expression and production of proteins and their phenotypic effects or exact roles in mastitis are not well defined.

### 2.4 Intracellular survival of *Staphylococcus aureus*

*S. aureus* can internalize into and multiply in different types of phagocytic and non-phagocytic cells (206). Viable *S. aureus* has been demonstrated in macrophages from milk samples of cows with mastitis (206). *S. aureus* can persist in the intracellular area of immune cells of different species (334, 335). However, the detailed molecular mechanisms of how *S. aureus* survives in the intracellular area are not fully defined. One of the mechanisms believed to be responsible for the intracellular survival of *S. aureus* is the induction of the formation of autophagy, which leads to the formation of autophagosomes that cannot bind to lysosomes to form autolysosomes that destroy *S. aureus* (334). Autophagy is a host defense mechanism or a eukaryotic cell’s homeostatic mechanism for survival during cellular stress and for destruction and clearance of intracellular pathogens (336, 337). It has been shown that infection of bovine phagocytic cells by *S. aureus* induces the formation of autophagy, and the autophagosomes increase the number of viable intracellular *S. aureus* (334). Other studies have also shown that *S. aureus* could utilize autophagy to survive in cells (338, 339). Similarly, autophagy was induced in bovine mammary epithelial cells challenged by *S. aureus,* but the autophagic flux was obstructed, leading to an increased number of intracellular *S. aureus* (340). Inhibition of the formation of autophagosomes in bovine mammary epithelial cells improved the clearance of intracellular *S. aureus,* whereas enhancing the formation of autophagosomes with the inhibition of the degradation of the autolysosomes increased the number of *S. aureus* inside bovine mammary epithelial cells (340). Several pathogens have developed mechanisms to avoid or even utilize the autophagic process to persist and multiply in host cells (341). Some studies show that *S. aureus* internalized into intracellular areas and remains in a membrane-bound vacuole, being converted to small colony variants (SCVs) with atypical small morphology and dormant biochemical properties, enabling it to survive in intracellular areas protected from host defenses and effects of antimicrobial drugs (342, 343) in dairy cows with a history of chronic intramammary *S. aureus* infection (78, 344). Cytotoxic *S. aureus* strains internalized into epithelial cells and could exit from the phagosome into the cytosol, where they multiplied and employed staphylococcal cysteine proteases and induced host cell death (219). The authors also reported the presence of serotype eight capsular polysaccharides (Cap), adenosine synthase A (AdsA), cysteine proteases (staphopain B SspB, and staphopain C, SspC), which are responsible for biofilm production and intracellular survival (219, 225) in all isolates. *S. aureus* can switch its phenotypes between wild types and small colony variants and survive inside cells, causing persistent intramammary colonization leading to recurrent bovine mastitis.

## 3 Host defense against staphylococcal mastitis

### 3.1 Natural defense

#### 3.1.1 Physical barriers

The teat canal opening is closed by the smooth muscle sphincter or Rosette of Furstenberg (345, 346) and keratin plug, a wax-like product of stratified squamous epithelial cells in the teat canal (346). Keratin contains bacteriostatic fatty acids (347) and fibrous structural proteins (348–350). Fibrous proteins are produced by stratified squamous epithelial cells in the teat canal that bind to bacteria and induce changes in the cell wall that make them prone to osmotic pressure and death (346). Fibrous proteins inhibit *Streptococcus agalactiae* and *Staphylococcus aureus* (351) and are functionally similar to bovine neutrophils (352). Keratin plug breakage (353) or interference with keratin formation due to damage by a faulty milking machine (354) increases bacterial invasion and colonization (355). After milking, the teat canal remains open for about 2 h, and during this time, bacteria can enter the intramammary area (356–358).

Despite the presence of these physical barriers (sphincter muscle and keratin plug) and bacteriostatic fatty acids and scleroproteins, *S. aureus* can gain access to intramammary areas and cause IMI during dry and lactation periods, as confirmed by previous studies (160, 359) or remain alive for several days after being infused a few millimeters inside the teat canal (360–362). The contaminant microorganisms from milking liners or milkers’ hands can be propelled from the open teat area into the teat cistern by fluctuating milking machine pressure, which is believed to be the major way for the spread of contagious mastitis pathogens to the proximal part of the mammary glands (363).

#### 3.1.2 Mammary gland microbiome and long non-coding RNA (lncRNA) and microRNA (miRNA) in milk

Mastitis has long been associated with a variety of bacterial pathogens. However, approximately 10–40% of clinical mastitis cases yield “no significant growth” following routine bacteriologic culture. Current advances in sequencing technology allow the comparison of culture-negative quarters with clinical mastitis to that of clinically normal quarters (364). Recent sequencing studies have revealed that milk, once considered sterile, is actually home to a complex microbial community with great diversity (365). Normal milk hosts a diverse community of non-culturable bacteria. Several bacterial species were differentially abundant in the clinical mastitis samples compared to the control quarters. Some culture-negative clinical cases have demonstrated almost 100% abundance of some species (e.g., Mycoplasma sp.). Further investigation is needed to determine the roles of mammary gland microflora in SCC and the physiologic basis for these associations, as well as to evaluate the microbial dynamics during and following IMI. Given the increasing recognition of the complex and important role of microbiota in host health, an analysis of the microbiota under health and disease conditions would provide important information on the role of microbiota in udder health.

Long non-coding RNA (lncRNA) is a novel endogenous non-coding RNA molecule with a length of more than 200 nucleotides (nt) (366) that is involved in transcriptional and epigenetic regulation of human and animal genes (367, 368). lncRNAs are emerging as critical regulators of gene expression in the immune system (369). lncRNAs are expressed in a highly lineage-specific manner and control the differentiation and function of innate and adaptive immune cell types (369). In the body’s immune response, lncRNAs regulate the occurrence and development of various inflammatory diseases, including bovine mastitis. Wang et al. (370) identified differentially expressed lncRNAs in the mammary epithelial cells induced by *E. coli* and *S. aureus* using high-throughput sequencing. Currently, only four lncRNAs —lncRNA H19 (371–373), lncRNA TUB (370), lncRNA XIST (374), and LRRC75A-AS1 (375)—have been studied with respect to their role in bovine mastitis.

### 3.2 Immunity

Mammary gland infection by bacteria or fungi induces immune responses (376, 377). Two types of immunity are induced by infection: innate and adaptive (378). Both are very important for the immune-mediated control of invading pathogens in mammary glands.

#### 3.2.1 Innate immunity

The skin, teat sphincter, and teat canal membranes serve as the first line of defense. Once the physical barriers are compromised, innate immunity gets involved. The teat canal tissue expresses toll-like receptors (TLRs) and secretes cytokines and antimicrobial peptides (379, 380). Innate immunity is divided into cellular (Leukocytes: neutrophils, macrophages, lymphocytes, and mammary epithelial cells) and humoral (Lactoferrin, transferrin, lysozyme, lactoperoxidase, and myeloperoxidase, complement systems, cytokines, chemokines, host defense peptides) components (346, 381).

##### 3.2.1.1 Cellular

**Neutrophils** are the most abundant (80%) leukocytes during IMI, and they are recruited by innate immunity (382). Neutrophils are recruited to the site of infection following chemical signals (chemoattractants), which include C5a, C3a, and IL-8 from the infection site (383, 384). The production of chemoattractants can be triggered by staphylococcal lipoteichoic acid (LTA) that attracts neutrophils and monocytes to the infection sites (385). Bone marrow produces neutrophils, which enter blood circulation and circulate through blood under normal circumstances. When there is IMI, their production is increased, and they are recruited from blood circulation into the infection site following chemoattractants. At high concentrations of chemoattractants, neutrophils slow down their movements through blood by binding with their cell surface receptor to the ligand on endothelial surfaces and move out of the blood into the infection site by squeezing themselves (diapedesis) between endothelial cells (386).

Some *S. aureus* strains can avoid getting killed by neutrophils (387, 388) and stay inside phagocytic cells. In that case, the natural killer cells (NK) or cytotoxic T cells kill infected phagocytic cells, releasing *S. aureus* for another possibility of killing by phagocytic cells (389). If *S. aureus* is not controlled by innate immunity, adaptive immunity takes over the battle through antibodies specifically produced against *S. aureus* that bind to bacteria and clear them by opsonophagocytic killing of phagocytic cells. Previous studies (390–392) have demonstrated that IL-8 is the most important chemoattractant for neutrophils-based quick response. A quick and effective cellular response is required to control *S. aureus* IMI from developing into mastitis.

##### 3.2.1.2 Humoral

**Lactoferrin** deprives the infected area of iron, leading to oxidative stress, preventing bacterial multiplication and growth, and assisting the survival of host cells (393).

**The complement system** is a series of proteolytic processes involving 30 plasma and cell surface proteins that lead to the production of proinflammatory mediators, opsonins, and membrane attack complexes (394). There are three complement pathways that clear invading pathogens. These include (1) classical, (2) lectin, and (3) alternative systems (395). The C3a and C5a are anaphylatoxins that induce histamine, vasodilation, and inflammation to eliminate or remove pathogens (395). The membrane attack complex (MAC) breaks holes, or pores, into the invading bacteria’s cell membranes, causing irreparable damage (396).

**Antimicrobial peptides (AMPs)** are small peptides of 10 to 60 amino acids that are commonly present in animals (mammals, amphibians, insects, aquatic), plants, and microorganisms with a broad spectrum of antimicrobial activity on bacteria, fungi, parasites, and viruses (397, 398). Almost all AMPS are cationic, but some are anionic (350, 398).

Antimicrobial peptides are also produced by different tissue cells, such as PMNs, macrophages, and mucosal epithelial cells. Antimicrobial peptides that are present in cattle are defensins, cathelicidins, and anionic peptides (399). Domestic animals have many cationic AMPS and a few anionic AMPS (400). Other mammalian AMPS are histatins (401) and dermcidin (402). Antimicrobial peptides kill microbes by different mechanisms, including the induction of ion channel formation (e.g., defensins) (403) and flocculation of intracellular contents (e.g., anionic peptides) (404), thereby affecting transport and energy metabolism (e.g., bactenecins) (405, 406).

β-defensins are AMPS mainly produced by polymorphonuclear cells (407–409). Lipopolysaccharide (LPS) and lipoteichoic acid (LTA) induce the production of β-defensins by mammary epithelial cells (410).

**Type 3 immunity** – Mastitis is usually caused by bacterial infections such as streptococci, staphylococci, and coliform bacteria, which is characterized by massive recruitment of neutrophils into mammary glands. Consequently, cell-mediated immunity, especially type 3 immunity, is the most likely intramammary defense mechanism. However, this mechanism is not well investigated. Efforts toward improving intramammary immunity against bacterial mastitis pathogens through better vaccine design that enhances type 3 immunity can be beneficial in controlling and understanding effective intramammary immunity.

Recent studies have shown that both innate and adaptive cell-mediated type 3 effector immunity have the capability to function as effectors on epithelial and mucosal surfaces (411, 412). Type 3 immunity is characterized by the recruitment of neutrophils, production of antimicrobial defenses by epithelial cells, involvement of type 3 innate lymphoid cells (ILC3s), expression of cytokines (IL-17A, IL-17F, IL-22), and transcription factors (retinoic acid-related orphan receptors γt and α -Rorγt and Rorα) (412, 413). Cells that are responsible for type 3 immunity include ILC3s, γδ T cells, CD4+ helper T cells (Th17), and CD8+ cytotoxic T cells (Tc17) (414). IL-17A-producing CD4+ cells were isolated from ruminants, and the Th17 cells were purified and cultured *in vitro* (258, 415, 416). The CD4 and CD8 lymphocytes with characteristic features of memory lymphocytes were detected in the milk from healthy and infected udder quarters (392, 417). The RORγt-expressing and IL-17A-producing CD4+ T cells were detected in mouse mammary glands, but CD8+ T cells expressing RORγt were not yet detected (418, 419). The innate immune system receptors [e.g., Toll-like receptors (TLR); TLR1, TLR2, TLR3, TLR4, and dectin-1] expressing T 17 cells and γδ T cells that can respond to mammary associated molecular patterns (MAMPs) were detected (420, 421). They could also secrete IL17A and IL-22 without interacting with the T-cell receptor (TCR) in the presence of IL-1β and IL-23. Bovine WC1+ γδ T cells, CD4+ (T17), and CD8+ T cells produce IL-17A (415, 416, 422, 423). In the peripheral tissues, a majority of the bovine γδ T cells are WC1- and functionally different from the WC1+ cells (420). Specific γδ T cells were shown to be recruited into milk during infection (391, 424). The ILC3 reside in the parenchymal tissues and mucosal-epithelial surfaces, where they function as effectors of cell-mediated innate immunity to protect against infection by pathogens and regulate inflammation and homeostasis (425). Bovine ILCs have not been detected yet, but human and mice ILCs have been shown to exist, and human ILCs can respond to pathogen-associated molecular patterns (PAMPs), whereas mice ILCs cannot. The ILC3 are stimulated by IL-23 and IL-1α or IL-1β and produce effectors such as IL17A, IL-17F and IL-22 (425).

#### 3.2.2 Adaptive immunity

Adaptive (acquired) immunity is a more advanced immune system that exists in higher vertebrates (426, 427). It consists of humoral (immunoglobulin-mediated) and cellular (cell-mediated) immunity. The innate immunity creates the basis for the induction of adaptive immunity during phagocytosis, processing, and presenting of antigens of infecting staphylococci to the immune system (428). Due to this process, adaptive immunity takes approximately a week to respond to an infecting pathogen. Adaptive immunity involves antigen processing and presentation by antigen-presenting cells (APCs). An antigen can be processed and presented to the naïve T cells circulating in the body by binding to major histocompatibility molecule I (MHC-I) or (MHC-II). All nucleated body cells can process and present antigens generated in the intracellular area coupled to MHC-I molecule, but only professional antigen-presenting cells can process and present extracellular antigens coupled to MHC-II molecules. There are three types of professional antigen-presenting cells. These are macrophages, B-lymphocytes, and dendritic cells. The mature naïve T cells released from the thymus and circulating in the blood frequently exit from blood circulation into regional lymph nodes at high endothelial venules where they bind to foreign antigen attached to MHC-II by its T cell receptor (TCR) and become activated T helper cells (e.g., Th1 or Th2 or Th17). The helper T cells activate B-cells to become antibody-producing plasma cells or activate other T-cells to become cytotoxic effector cells depending on the type and location of antigen in the body (429).

To prevent the body from future attack by the same etiological agent, the adaptive immune system produces memory T cells (430) and B cells. For antigens generated in the intracellular area, the helper T cells activate CD8+ T cells to become effector cytotoxic T cells that kill infected cells. For extracellular pathogens, the T helper cells activate B-cells to become antibody-producing plasma cells. The antibody binds to the pathogen and leads to its removal by opsonophagocytic mechanism (431) or block bacterial binding to host tissue surface receptors (382).

Adaptive immunity produces antibodies or activated cytotoxic T cells that remove pathogens and memory cells (T and B cells) that keep the information about a specific pathogen for quicker response in case of future attack by the same pathogen.

## 4 Host-pathogen-environment interactions as risk factors for staphylococcal mastitis

There are many host, pathogen, and environmental risk factors for mastitis. The host risk factors include age/parity, lactation stage, somatic cell count, heredity, anatomical structure of the udder and teat, local defense mechanisms or immune competence, colonization with less pathogenic pathogens, and the presence of other diseases (432). Parity is one factor; a cow on its third lactation or greater is prone to developing clinical mastitis (433). An increase in the number of lactations increases the chance of exposure to mastitis pathogens and deterioration of previous infections (433). Cows are more likely to develop clinical mastitis (CM) during the first 30 days postpartum, with >50% of cases of mastitis occurring during this period than the remaining days of lactation (434). However, 80% of the CM cases occurring after 30 DIM were due to new IMI (434).

Pathogen risk factors include the type of pathogen (staphylococci), volume, genotype of the strain (74, 435–438), ability to form biofilm (439–441), formation of small colony variant (78, 343), frequency of exposure, methicillin-resistant *S. aureus* (MRSA) (442), attachment and internalization ability (79, 271), and resistance to antimicrobials (443, 444). The type of bacterial species affects infection duration, severity, treatment outcomes, and milk yield. More than 50% of recurring CM cases are due to the same pathogen that caused mastitis in the same animal previously (445).

The environmental and/or managemental risk factors include faulty milking machines, udder injury, hygiene, climate, nutrition, the season of the year, housing, and biosecurity measures (446). The prevalence of mastitis can be affected by post-milking teat dipping, clean and dry bedding, cleaning teat orifice with antiseptic solution before giving intramammary infusion, milking cows with CM last, good maintenance for the milking machine, preventing udder trauma, and climate. Warm and humid climates support the multiplication and growth of bacteria and the risk of IMI and mastitis (446).

*Staphylococcus* species vary in their ability to induce inflammatory reactions in the mammary glands, and SCC with the highest counts is usually caused by *S. aureus*. However, other NAS species such as *S. chromogenes*, *S. hyicus*, *S. agnetis*, *S. simulans*, and *S. xylosus* have also been reported to cause increased SCC similar to *S. aureus* (87, 119). *S. simulans*, *S. agnetis,* and *S. hyicus* cause robust inflammatory responses (101, 104, 105, 107). *S. simulans* is more resistant to phagocytic killing, whereas *S. chromogenes* can be easily phagocytosed and killed. *S. simulans* is usually isolated from the milk of cows with mastitis (101, 104–106). In field studies, *S. simulans* caused more clinical mastitis than others (101, 106), and experimentally-induced mastitis by *S. simulans* caused a stronger inflammatory response than *S. epidermidis* (121). Similarly, another study found that *S. chromogenes* originally isolated from milk with mastitis induced more inflammatory reactions than *S. chromogenes* from the teat apex (122). In another study, *S. epidermidis* and *S. haemolyticus* caused high SCC (123). In some studies, a slight increase above 100,000 cells/mL was reported for quarters infected with NAS (109, 124), whereas in another study, SCC varied from as low as 70,000 cells/mL to as high as 123,000 depending on the species of NAS involved (20). Some NAS species (*S. agnetis*, *S. hyicus*, *S. simulans*) caused clinical mastitis more frequently than others (101, 104, 105), whereas some others (e.g., *S. epidermidis*) caused mild inflammatory responses than *S. simulans* (121). Based on molecular data, *S. simulans* was usually isolated from milk with mastitis, but *S. chromogenes* can be associated with subclinical mastitis as well as skin microbiota (24, 100). Despite observed differences in the opsonophagocytic killing between *S. simulans* and *S. chromogenes,* both can usually exist in the mammary glands throughout lactation and be responsible for increased SCC (103, 126). Under controlled experimental infection (121), the majority of *S. simulans* induced chronic infection. *S. agnetis* was more phagocytosed by murine macrophages than *S. simulans* (125) but more resistant to killing, similar to *S. simulans* and *S. aureus* (125). *S. aureus* usually caused subclinical mastitis that often became chronic with a moderate increase in milk SCC. NAS occasionally caused clinical mastitis with SCC, usually ranging in the low to moderate increase, but could cause significantly increased high SCC (22).

The pathogenesis mechanisms responsible for the differences between NAS and *S. aureus* are still unknown and need further investigation. In some studies, *S. simulans* was different from other NAS in opsonophagocytic killing (125). However, other studies that used neutrophils instead of macrophages, which were recruited to the mammary gland after macrophages initiated an inflammatory response, reported significant differences in opsonophagocytic killing among *S. aureus* strains (447). All observed differences were not correlated with the type of mastitis (clinical or subclinical) (125). There was a difference in the opsonophagocytic killing of some NAS by murine macrophages (125). Staphylococci can resist opsonophagocytic killing by the formation of capsules and other extracellular polysaccharides (130–132). There are differences among NAS isolates in their susceptibility to opsonophagocytic killing by macrophages (125). These differences could be due to yet unknown novel virulence factors. Therefore, further investigation is required.

## 5 Pathogenesis of staphylococcal mastitis and clinical manifestation

*S. aureus* and NAS enter the intramammary area either by progressive colonization from the teat apex or propelled into the intramammary area during milking machine vacuum fluctuations (80). *Staphylococcus aureus* binds to the α-5β1 integrin on the mammary epithelial cell surface through fibronectin-binding proteins (FnBPs) (448). The presence of FnBP is vital for adherence, but its expression may vary with *S. aureus* strains (448). This initial adherence leads to actin polymerization, cytoskeleton formation, and entry of bacterium into the host cell (448).

Staphylococcal mastitis affects physical and chemical properties and microbial status in milk due to pathological changes in the udder tissue (449). These changes in milk and gland tissue are characterized by visible abnormal local inflammatory signs in milk and gland tissue or systemically in the animal body (15). *Staphylococcus aureus* mastitis can manifest as peracute, acute, or chronic clinical forms or subclinical forms. Subclinical *S. aureus* mastitis is the most common udder infection in dairy cows (213), but *S. aureus* is also one of the most common causes of clinical mastitis in dairy cows (450, 451). Clinical *S. aureus* mastitis varies from mild changes in milk to peracute gangrenous mastitis with severe systemic manifestations and death of infected cows (213). Severe cases occur occasionally in dairy cows (452–454). Severe peracute gangrenous mastitis has been reported in other species, including sheep (217), goats (455), rabbits (456), and humans (457). Clinical *S. aureus* mastitis is characterized by swollen, red, hot, and painful udder with total loss or reduced milk yield (359). Subclinical *S. aureus* mastitis does not show clinically visible abnormal inflammatory changes in the milk and/or gland tissues but reduces milk yield and quality. The occurrence of SCM is 15–40 times higher than CM (458). *S. aureus* mastitis is usually subclinical and chronic, with low cure rates even with antibiotic treatment (89).

Acute and peracute *S. aureus* mastitis is manifested by sudden onset with the swollen udder, fever, and purulent inflammation. The sudden onset during the first few days after parturition may develop into gangrene and is highly fatal. Local clinical mastitis may develop into systemic acute or peracute mastitis manifested by increased temperature, pulse, and respiratory rates, anorexia, toxemia, muscle weakness, ruminal stasis, and dehydration (459). Chronic *S. aureus* mastitis is manifested by high SCC, gradual inflammatory process, necrosis, fibrosis, atrophy of the udder, decrease in milk production, occasional clots in milk, and watery milk. Chronically infected cows must be culled before the infection spreads through the whole herd (215).

## 6 Diagnosis of staphylococcal mastitis

### 6.1 Clinical signs

Clinical mastitis causes damage to the blood-milk barrier in the gland epithelial lining and breaks tight junctions, causing the leakage of blood, cells, and other extracellular fluid components (460) into milk and udder tissue, resulting in visible abnormal changes in milk and mammary gland tissue as clinical signs (460). Leukocytes, especially neutrophils, are recruited to the gland to fight off infection. The fight results in dead bacteria, mammary gland cells, and tissue forming purulent inflammatory fluid or pus that are usually seen when foremilk is stripped out prior to milking. The influx of fluid and white blood cells results in a swollen gland, and the increased flow of blood to the infected area causes redness/hyperemia and increased heat on the gland tissue surface. The gland tissue becomes painful to touch due to increased pressure on local nerve fibers, and the death of milk-producing cells leads to decreased or loss of milk yield, which altogether constitute cardinal signs of inflammation or mastitis (460). Most studies consider NAS species as minor pathogens that cause only a slight increase in SCC and mild clinical mastitis (CM) (96, 142–145). However, differences among species are not well defined and understood.

### 6.2 Bacteriological culture

Bacteriological culture is a good method for diagnosing *S. aureus* IMI. However, because of the cyclical shedding of *S. aureus* through milk, more than two consecutive milk samples are required to increase the sensitivity of the culture result (461, 462). Individual quarter milk culture has higher sensitivity (463) than composite milk culture, but the sensitivity of bacterial culture is affected by the type of sample (individual or composite), volume, and time interval of repeated samplings. Individual quarter milk sampling at one-day intervals with 0.1 mL volume culturing separately is expected to have sensitivities of 90 to 95%, whereas individual quarter milk sampling at three or four-day intervals with 0.1 mL volume culturing separately is expected to have sensitivities of 94 to 99%. Daily individual quarter milk culturing separately provides a sensitivity of 97% and a specificity of 97 to 100%.

*S. aureus* in milk samples from clinical mastitis ranges between 10^4^ and 10^5^ CFU /mL, but only one colony needs to be positive (464). However, *S. aureus* and NAS can be isolated from udder quarter milk samples of dairy cows without an increase in SCC (465, 466). Subclinical and clinical mastitis cases due to NAS had 10^3^–10^4^ and 10^5^–10^6^ CFU/mL of bacterial counts, respectively (18). A milk sample containing at least 10 NAS or 1,000 CFU/mL of milk with SCC > 100,000 cells/mL is considered subclinical mastitis. Composite milk culture increases the number of false-negative results than individual quarter milk culture; however, culturing 500 μL than 10 μL increases sensitivity. Reports from different studies indicated that freezing milk samples had no effect on *S. aureus* count or increased count because of cell death and release of intracellular bacteria (467–469). Staphylococci are differentiated from other gram-positive cocci, especially streptococci, by positive coagulase and catalase tests. *S. aureus* may cause double hemolysis on blood agar characterized by an outer zone of incomplete hemolysis due to β-hemolysin with an inner zone of complete hemolysis due to α-hemolysin (470, 471), but the production of hemolysins varies with strains (471). A tube coagulase test is an important test, and *S. aureus* is coagulase-positive with 100% specificity within 24 h. Coagulase-positive *Staphylococcus* species can be differentiated by matrix-assisted laser desorption ionization time-of-flight mass spectrometry (MALDI-TOF MS) or inoculating a colony from blood agar plate with hemolysis after 24 h into *S. aureus* CHROMagar plates; mauve to rose colonies (470) is a positive diagnosis for *S. aureus*. NAS isolation and individual pure colonies can be obtained following National Mastitis Council Guidelines (472), and each pure colony is identified at the species level by MALDI-TOF MS (96, 473).

### 6.3 MALDI-TOF MS

The MS principle involves ionizing chemical compounds to generate charged molecules and measure their mass-to-charge ratio. Such molecular “signatures” can be used for rapid bacterial identification from isolated colonies. It can differentiate *S. aureus* from the other coagulase-positive *Staphylococcus* species (474–476). In a previous study testing 152 staphylococcal species, 99.3% were identified correctly (477). Another study found that the MALDI-TOF MS achieved 100% specificity and sensitivity when characterizing coagulase-positive and negative strains of staphylococcal species isolates (478). The limitation of MALDI-TOF MS is the lack of non-clinical isolates in the comprehensive database for comparison, for example, cases of NAS mastitis (474).

### 6.4 Somatic cell count

A healthy individual quarter has SCC < 100,000 cells/mL, and an individual quarter infected with minor pathogens has SCC > 100,000 cells/mL, whereas an individual quarter infected with major pathogens has SCC > 350,000 cells /mL. If composite milk (from 4 quarters of a cow) SCC < 200,000 cells/mL, milk production loss is not expected or minimal, but a few quarters may have an infection (479, 480). The bulk tank SCC threshold of ≤200,000 cells/mL of milk is used to determine high-quality milk that qualifies for premium milk sale price. The International Dairy Federation considers an SCC > 200,000 cells/mL as a case of subclinical mastitis regardless of any determination of the presence of microorganisms. In addition to monitoring SCC at bulk tank milk level to determine high milk quality that can be sold at premium prices, individual cow SCC is used to identify and treat or segregate specific subclinically infected animals to continue to ensure high milk quality and low transmission of pathogens during milking.

Different indirect testing methods can detect the presence of an inflammatory response in milk samples. These include the Sodium Lauryl Sulphate Test (SLST), California Mastitis Test (CMT) (481), White Side Test (WST), electric conductivity (EC), pH Multistix strips, detection of enzymes, Tanuchek kits, DeLaval cell Counter (DCC), flow cytometry, and Surf Field Mastitis Test (SFMT).

## 7 Control of staphylococcal mastitis

### 7.1 Management

The National Institute for Research into Dairying developed a five-point mastitis control measure in England (482), and later, these measures were adopted by the National Mastitis Council (NMC) as a five-point mastitis management program or Five-Point Plan for the Control of Contagious Mastitis (483–485). The Five-Point Plan comprises (1) post-milking teat dipping in antiseptic solutions, (2) antibiotic dry cow therapy at the end of each lactation, (3) treatment of clinical cases, (4) culling of cows with chronic mastitis, and (5) proper maintenance of the milking machine to maintain stable teat end vacuum pressure. After implementing this plan, infection rates decreased by up to 50%, and cow-to-cow transmission also decreased gradually. The NMC improved the five-point plan to a ten-point plan by adding five additional measures such as (6) setting goals for udder health, (7) keeping cows in a clean, dry, suitable environment, (8) good record keeping, (9) regular monitoring of udder health status, and (10) periodic review of the mastitis control program. Current *S. aureus* control measures include maintaining healthy teat condition, pre-milking teat dipping in antiseptic solution and drying, using disposable gloves during milking, keeping the milking machine in good condition, post-milking teat dipping in antiseptic solution, dry cow therapy, cull chronic cases, and milking infected cows last. Teat dipping in antiseptic solution pre- and post-milking decreased new IMI by 50 to 65% compared to control cows without dipping teats (486).

### 7.2 Use of antimicrobial drugs

#### 7.2.1 Therapeutic

Prudent antimicrobial drug use and antibiotic stewardship in dairy farms are strongly recommended to reduce the development of antimicrobial resistance (AMR). The chance of cure by antibiotic treatment depends on treatment plans for cows and pathogen-related risk factors (487, 488); however, these factors are not considered during the treatment of *S. aureus* mastitis (30). The cure rates for subclinical *S. aureus* mastitis range from 4 to 92% (489, 490). The chance of cure of an infected quarter decreased when SCC increased (> 250,000 cells/mL) (491), cow aged, another quarter of the cow had IMI, hindquarter infected, and high prevalence of *S. aureus* IMI before drying off (30).

Management-based mastitis control measures have been developed and implemented with mild success in reducing contagious bacteria such as *S. aureus* and *S. agalactiae* (26–28) but limited success due to application disparities across mastitis management (29).

The cure rate of *S. aureus* mastitis with intramammary treatment during lactation or at dry-off is poor (30, 173–175) and rarely exceeds 50%. *S. aureus* IMI usually exists throughout the lactation period due to limited anti-microbial drug access to the *S. aureus* in the purulent inflammatory fluids or formation of micro-abscess and fibrosis (492–494), *S. aureus* formation of L-forms (495, 496) or small colony variant (78), β-lactamase production (488, 497), survival of *S. aureus* in the intracellular area of phagocytic cells (76, 498) and internalization into mammary epithelial cells (79, 271, 499). The selection of antibiotics for treatment based on *in vitro* susceptibility testing may not be effective under *in vivo* conditions. However, for *S. aureus* mastitis cases of less than 2 weeks’ time, an *in vitro* susceptibility test can be used as a predictor of cure, but not for chronic cases of mastitis (490). The importance of antibiotic susceptibility testing for the treatment of clinical mastitis is arguable (500), yet the majority agree that it is better to do susceptibility testing (30) than treat without testing.

Antibiotics approved for use in dairy cattle for treatment and the prevention of mastitis and other dairy cattle diseases (bovine respiratory diseases, feet infection, metritis, diarrhea, or scours) include cephalosporins, fluoroquinolones, aminoglycosides, penicillin, sulfonamides, macrolides, amphenicols, tetracyclines, and lincosamides (501, 502). In the US, there are seven approved intramammary (IMM) antimicrobial drugs (502, 503). These include lincosamides (pirlimycin) and beta-lactams which include cephapirin (first-generation cephalosporin, 1GC), ceftiofur (third-generation cephalosporins, 3GC), aminopenicillins (amoxicillin and hetacillin), penicillin G, and penicillinase-resistant penicillin (cloxacillin) (376, 502).

Clavulanic acid with amoxicillin or cloxacillin with ampicillin overcomes β-lactamase resistance, making them antimicrobial drugs of choice for intramammary formulation. The 1GC 3GC and erythromycin are effective against β-lactamases-producing staphylococci.

Antibiotic treatment of subclinical *S. aureus* mastitis during lactation is not economical because of the low cure rate, milk disposal during treatment, and lack of increased milk yield after treatment (30). Intramammary infusion of long-acting antibiotics at drying off (dry cow therapy) is more effective, with a 40–70% successful clearance rate (504). The cure rate of a lactating cow with antibiotic treatment depends on the length of infection, number of udder quarters infected, type of quarter (hind or front) infected, strain of *S. aureus*, immunity of the cow, type of antibiotic used for treatment, and length of treatment with better cure rate and longer treatment (489). Current recommendations are to use both IMM and parenteral antibiotics or treat only with IMM for 4–8 days. Penicillin G is the antibiotic of choice for penicillin-sensitive *S. aureus* strains. The IMM of pirlimycin is effective when administered for 8 days (505). Extended treatment with pirlimycin decreased transmission and clinical mastitis in the herd (505).

A previous study on subclinical *S. aureus* mastitis treatment with IMM antibiotics showed no difference between treated and untreated controls with bacterial cure rates of 65, 47, and 43% for erythromycin and penicillin, cloxacillin, and amoxicillin and cephapirin, respectively, (89). Treatment of clinical *S. aureus* mastitis with extended cefquinome IMM improved clinical cures from 60 to 84% but did not change bacterial cures (491). The treatment of subclinical *S. aureus* mastitis with simultaneous IMM of amoxicillin and intramuscular injection of procaine penicillin G achieved a cure rate of 50% (506).

Staphylococci are known to become resistant to several antibiotics, including methicillin resistance, which is important for public health (163, 164). Methicillin-resistant *Staphylococcus aureus* (MRSA) infection can only be treated with limited antibiotics and needs long-term treatments (163, 165–167). MRSA infection is zoonotic (168), and continuous antimicrobial susceptibility surveillance is crucial to control the transmission of this strain from animal production to humans and vice versa (169). They may transfer resistance traits to *S. aureus* or other bacteria, resulting in the emergence of multidrug-resistant strains (94, 135). The prevalence of infection by these groups of bacteria is on the rise mainly due to the spread of resistance to antimicrobial drugs among these groups (135). The most frequently seen resistance among staphylococci is resistance due to the production of β-lactamases, with more common production among subclinical non-aureus staphylococci isolates than clinical isolates (170). They exhibit resistance to multiple classes of antimicrobial drugs (32, 171, 172).

#### 7.2.2 Prophylactic

In general, under an ideal dairy farming situation, cows are in lactation for about 300 days, and the dry period is about 60 days. Dairy cows are prone to IMI during the first 2 weeks of the dry period and during the transition period (507–509). The risk of IMI is high during the first 2 weeks of the dry period because of increased colonization of teat skin by bacteria due to the absence of pre- and post-milking teat dip in antiseptic solutions known to reduce bacterial colonization and IMI. During the transition period, dairy cows experience various metabolic, immunological, and physiological changes, increasing the risk of periparturient diseases (510). The high risk of IMI during the transition period is associated with parturition-inducing immunosuppressive hormones (e.g., cortisol), negative energy balance, and parturition-related stress (508).

In general, IMI during the dry period is expected to be low due to the involution and closure of the teat opening by the keratin plug in the teat canal. However, teat canal closure after drying off varies from animal to animal (511). Some bacteria may enter into the intramammary area by crossing the keratin plug or when the keratin plug is broken by intramammary infusion. Dry cow therapy (DCT) has been used as the major preventive tool for new IMI, as well as to cure IMI or subclinical mastitis established during the previous lactation (511, 512). Additional benefits of DCT include no milk disposal and treatment with antibiotics during the dry period to achieve high bacteriologic cure rates. There are two kinds of DCT. These are blanket and selective DCT. Blanket dry cow therapy (BDCT) is an IMM of long-acting antibiotic into all quarters of lactating cows on farms at drying off. The BDCT is the most common form of usage in over 90% of dairy farms in the US (513). According to the US Department of Agriculture (USDA) survey results, 85% of conventional dairy farms use BDCT (514), which is estimated to account for one-third of the total antibiotics used on conventional farms in the US (515). According to the 2013 USDA National Animal Health Monitoring System (NAHMS) survey, antibiotics used for the treatment of mastitis accounted for 85.4% of antibiotics used on US dairy farms (USDA, 2016). BDCT is of growing concern because this practice exposes healthy animals to antimicrobials, allowing for antimicrobial selection pressure on commensal and opportunistic bacteria to develop AMR.

Selective dry cow therapy (SDCT) selectively treats only quarters of an infection during drying-off. Despite decreasing antibiotic usage, SDCT is only applied in 10% of US dairy operations (501), and the risk of missing IMI exists when compared to BDCT (114, 516, 517). The concern is the increased risk of IMI could influence herd health and profitability (518, 519). SDCT needs to be evaluated in great detail before fully implementing it across dairy operations. However, with growing concern about the use of antibiotics in food animals, BDCT is being extensively reviewed and has motivated research into alternative disease control measures (520). Finding alternatives, such as effective vaccines, for preventive antibiotics use at dry-off is key in controlling mastitis and easing concerns of AMR.

Alternatives to antibiotics, such as internal teat sealants, are shown to reduce IMI during the dry period (521) and reduce new IMI after calving when used with or without antibiotics (522). Another alternative is boosting the nutritional supplement of dairy cows with diet or supplementation of feed with nutrients that boost the immune system. Well-known dietary ingredients in the dairy industry, vitamin E and selenium (Se), when fed daily, promote immune competency and reduce the duration of clinical mastitis (523, 524).

#### 7.2.3 Antimicrobial resistance

Specific antibiotic usage data are not available from dairy farms in the US, and it is not possible to know the exact amount of antibiotics used. Information on doses, frequency, duration, and diseases treated are also not known. However, the US Food and Drug Administration (FDA) report showed that more than 16,155 kg of medically important antimicrobial drugs intended for intramammary therapy were sold in 2019 (525). A previous review showed no widespread resistance among mastitis pathogens (444). However, some studies have shown that the treatment of mastitis with antibiotics is associated with AMR and changes in the diversity of mastitis pathogens (526, 527). Similarly, other studies (528) have established a positive association between antimicrobial drug use (pirlimycin, ampicillin, erythromycin, and tetracycline) and increased resistance among gram-positive mastitis pathogens. Another previous study (529) showed higher resistance among bacterial mastitis pathogens from conventional dairy farms (ampicillin, erythromycin, penicillin, and tetracycline) than organic dairy farms, indicating antibiotics usage increases antimicrobial resistance. Yet another study (33) on *S. aureus* isolates from cases of mastitis in East Tennessee showed that about 34.3% were resistant to at least one of the 10 tested antimicrobial drugs. The authors also indicated an increasing trend in AMR in *S. aureus* for some antimicrobials (e.g., tetracycline).

*S. aureus* resistance to penicillin is well known. Penicillin-resistant *S. aureus* decreased in the US between 1994 and 2001 (530, 531), but resistance levels differ considerably across countries (532, 533) and within a country. The prevalence of penicillin-resistant *S. aureus* isolates from bovine mastitis in the US ranged from 30 to 70% (66, 531, 532). *S. aureus* resistance to macrolides ranged from 14 to 17% based on phenotypic testing (534).

A study on 121 NAS isolates from cases of bovine mastitis found that methicillin resistance was commonly observed among some (*S. epidermidis* and *S. haemolyticus*) isolates (535). Multiple NAS isolates were positive for *mecA* or *mecC* gene located on the staphylococcal cassette chromosome mec (SCC*mec*) (243). The *mecA* and its variant *mecC* encode for methicillin resistance. However, the use of *mecA* solely as a methicillin resistance marker provided false positives due to the ancestor of *mecA* naturally occurring in NAS (536).

A study on *S. aureus* from Canada identified the major facilitator superfamily (MFS) of transporters such as *tet* (37), *NorA*, and *NorB* efflux genes in all isolates from bovine mastitis (220). AMR genes, such as the *mepA* gene, code for multidrug export protein MepA and its repressor *mepR*. Additionally, the *norA* gene coding for quinolone resistance protein NorA and its regulators *arlS* (signal transduction histidine-protein kinase ArlS) and *arlR* (response regulator ArlR) were identified. Other genes detected in some isolates were *tet* (37) (tetracycline efflux MFS transporter), LmrS (major facilitator superfamily multidrug efflux pump), and *mgrA* (HTH-type transcriptional regulator MgrA, also known as NorR), which is a positive regulator for *norA* expression and repressor for *norB* and *tet38*. Finally, the *murA* (antibiotic-resistant murA transferase), *glpT* (antibiotic-resistant GlpT), and *fosB* (fosfomycin thiol transferase) were detected in some isolates (85).

### 7.3 Vaccines

Different vaccines were evaluated for the control of *S. aureus* mastitis in dairy cows (537). Vaccination with simultaneous antibiotic administration (36, 86, 538) and vaccination with autogenous vaccines (539) were shown to have some protective effects.

There is only one bacterin vaccine for *S. aureus* mastitis in the US, but recent efficacy studies concluded that it cannot be recommended to control *S. aureus* mastitis in the US because of its limited efficacy (34–36). Another bacterin vaccine is available in Europe for the control of mastitis caused by *S. aureus*, non-aureus staphylococci, and *E. coli*. Some efficacy studies with this bacterin vaccine concluded that it reduced the incidence, severity, and duration of mastitis (37–39), whereas others concluded that it did not confer a reduction in *S. aureus* mastitis (40–43).

In the US, based on reported efficacy results, there is no recommended vaccine for the control of *S. aureus* mastitis. Major obstacles to developing an effective *S. aureus* mastitis vaccine are the bacterial ability to survive in the intracellular area of phagocytic and non-phagocytic cells, strain variation, and variation of virulence factors and mechanisms with strain (540) that lead to different clinical symptoms in infected host (229). Further, the physiology of the mammary gland is such that the effector immunity is diluted with a large volume of milk that is removed two to three times daily (34, 541). An optimized vaccination regimen is critically required to achieve protection by an effective vaccine (542).

## 8 Priority research gaps that need to be addressed

Based on current literature, the following research gaps are evident and need to be addressed:

1. *Staphylococcus aureus* is a zoonotic bacteria that mainly causes human endovascular infections and bovine mastitis. Yet differences and similarities between human-adapted and bovine-adapted strains at cellular and molecular levels are not well defined, and further investigation and evaluation are needed to develop improved, knowledge-based control tools.
2. Non-aureus staphylococci comprise more than 50 species of diverse groups, including coagulase-negative, some coagulase-positive, and coagulase-variable staphylococci that vary in virulence, pathogenicity, and epidemiological distribution. Each species requires a focused, detailed study to understand its role in milk somatic cell count, the development of IMI and mastitis, and its contribution to normal milk microbiota and intramammary homeostasis.
3. Staphylococci are one of the major host-adapted opportunistic bacteria that live with a host for several decades and have several virulence factors. They are considered one of the intramammary microbiota in bovines, as determined by metagenomic sequencing at one time, but can cause IMI and mastitis at another time. However, there is a need for further investigation, especially from the perspective of innate and adaptive immunity, to understand the interactions between staphylococci and bovine hosts that allow them to remain opportunistic.
4. Current advances in sequencing technology allow the comparison of culture-negative quarters with clinical mastitis to that of clinically normal quarters. Sequencing studies reveal that normal milk hosts a diverse community of non-culturable bacteria. Several bacterial species were differentially abundant in the clinical mastitis samples compared to the control quarters. Some culture-negative clinical cases demonstrated almost 100% abundance of some species (e.g., *Mycoplasma* sp.). Further investigation is needed to determine the roles of mammary gland microflora in SCC and the physiologic basis for these associations.
5. *S. aureus* can internalize into and multiply in different types of phagocytic and non-phagocytic cells. In humans, *S. aureus* and NAS are also known to form biofilm *in vivo*, which is known to be responsible for infection resistance to the host’s immune response and antimicrobial drug treatment. However, the detailed molecular mechanisms of how *S. aureus* survives in the intracellular area of phagocytic and non-phagocytic cells and the role of biofilm in the pathogenesis of bovine mastitis need further investigation.

## 9 Conclusion

1. Mastitis is the most common disease of dairy cows and incurs huge economic losses in dairy farming worldwide. Bovine mastitis is an inflammation of the udder of dairy cows, usually caused by bacteria, which results in increased milk SCC and loss or reduced milk production. The most common bacterial etiology of mastitis are staphylococci, streptococci, and coliforms. Staphylococci are a major bacteria that cause mastitis and huge economic losses to dairy farms. According to recent reports, there are more than 60 valid species in the *Staphylococcus* genes, and each species varies in many aspects, including genetic makeup, pathogenicity, and ability to cause disease; even strains within species differ in their pathogenicity, virulence, and host adaptation. Because of these variations, each species of *Staphylococcus* should be considered different in its ability to cause mastitis, and appropriate control measures need to be designed based on the knowledge of each species. Current control measures for mastitis due to *S. aureus* and NAS are not fully effective. An improved understanding of virulence factors of dairy cows adapted strains of *S. aureus* and NAS, their pathogenesis, and host immunological responses is required to develop effective and sustainable non-antibiotic control tools such as vaccines, prophylactic therapy, and other innovative tools.

## Author contributions

OK: Conceptualization, Writing – original draft, Writing – review & editing. JV: Writing – review & editing.
